# An Isolated Mesenteric Presentation of a Nodal Peripheral T Cell Lymphoma with T Follicular Helper Cell Phenotype

**DOI:** 10.3390/hematolrep14040047

**Published:** 2022-11-15

**Authors:** Anna Keogh, Fiona Lynott, Antonios Papanicolau-Sengos, Mutaz Mohammed Nur, Aisling Spillane, Fiona Quinn, Ezzat ElHassadi, Elaine S. Jaffe, Richard Flavin

**Affiliations:** 1 Department of Histopathology, Saint James Hospital, D08 NHY1 Dublin, Ireland; 2 Department of Histopathology, Trinity College Dublin, D02 PN40 Dublin, Ireland; 3 Laboratory of Pathology, Center for Cancer Research, National Cancer Institute, National Institutes of Health, Bethesda, MD 20814, USA; 4 Department of Histopathology, University Hospital Waterford, X91 ER8E Waterford, Ireland; 5 Cancer Molecular Diagnostics Department, Saint James Hospital, D08 NHY1 Dublin, Ireland; 6 Department of Haematology, University Hospital Waterford, X91 ER8E Waterford, Ireland

**Keywords:** nodal PTCL with TFH phenotype 1, extra-nodal 2, mesenteric mass 3, T follicular helper cell origin 4

## Abstract

Nodal peripheral T cell lymphoma (PTCL) with T follicular helper (TFH) cell phenotype is a provisional entity added to the 2016 revised WHO classification of haematological malignancies. These lymphomas have an aggressive clinical course and respond poorly to conventional treatments. Distinct histological features have not been well described. Additionally, the minimum criteria for diagnosis is not well established but detection of at least two TFH markers in addition to CD4 is suggested to assign a TFH cell phenotype. Some pathological features of angioimmunoblastic T cell lymphoma (AITL) such as recurrent molecular alterations are commonly found. As the name suggests, these lymphomas are nodal in origin with patients presenting with widespread lymphadenopathy. We describe the first documented case of nodal PTCL with a TFH phenotype presenting as an isolated mesenteric mass with no nodal involvement.

## 1. Introduction

Multiple recent studies have identified a subset of PTCL, not otherwise specified (PTCL-NOS) as having a T follicular helper (TFH) cell phenotype with pathological features and recurrent genetic aberrations commonly seen in angioimmunoblastic T cell lymphoma (AITL) [1,2,3]. TFH cells are now established as the cell of origin of AITL and about 20% of PTCL-NOS [4]. The 2016 revised WHO classification, therefore, added two provisional entities of PTCL with a TFH phenotype, namely follicular T cell lymphoma (FTCL) and nodal PTCL with TFH phenotype.

Like PTCL-NOS, these lymphomas have an aggressive clinical course, respond poorly to conventional treatments, and have a dismal prognosis. The diagnosis is often challenging, requiring clinical, histological, and molecular input. In addition, the minimum criteria for assignment of a TFH cell phenotype is not well established; however, the detection of at least two (preferably three) TFH markers in addition to CD4 is suggested as diagnostic. TFH markers include PD-1, CD10, BCL6, CXCL13, ICOS, SAP, c-MAF, CD200 and CXCR5. Like AITL, these lymphomas have been described as nodal in origin with patients presenting with lymphadenopathy.

Here, we describe a nodal PTCL with a TFH phenotype that presented as an isolated mesenteric mass without nodal involvement which, to our knowledge, has not previously been documented in the literature.

## 2. Case Report

A 71-year-old male presented to the Emergency Department of a peripheral hospital with a two week history of weight loss, anorexia, abdominal pain, and fatigue. He had no night sweats or fevers. His past medical history was significant only for atrial fibrillation and heart failure with preserved ejection fraction (HFpEF); he had no history of any gastrointestinal illnesses or similar presentation.

**Figure 1:**
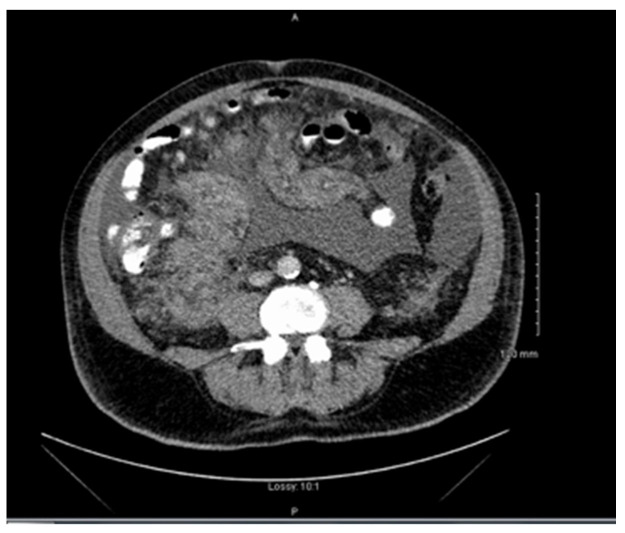
Computed tomography (CT) of the abdomen and pelvis showed a large mesenteric mass and a large volume ascites. There was no lymphadenopathy identified.

Clinical examination revealed abdominal distension secondary to large volume ascites. He had no palpable lymphadenopathy. Laboratory studies showed thrombocytopenia, hypercalcemia, and normal lactate dehydrogenase and liver function tests. Computed tomography (CT) revealed a large mesenteric mass (Figure 1). No adenopathy was identified. Ascitic fluid analysis did not yield a definitive diagnosis. The patient was transferred to our centre (a tertiary referral centre) for further work-up and management. By the time the patient was transferred to our centre, which does not have a PET CT on site, he was too unstable to be transferred to a PET scan centre.

A bone marrow aspirate demonstrated only reactive features, without evidence of an infiltrative process. Histopathological evaluation by means of biopsy of the mesenteric mass and a bone marrow trephine (BMT) were performed. The BMT showed no infiltrative disease. Initial biopsy results of the mesenteric mass had a wide differential, including a florid reactive/infective process and a lymphoproliferative disorder. It was subsequently sent for expert opinion.

While awaiting these results, the patient clinically deteriorated, with progressive ascites, anorexia, and fevers. An EBV viral load was found to be significantly elevated, >200,000 copies. CMV reactivation was also detected at this time.

Following discussion of the case at multiple multidisciplinary meetings (MDT), surgery was not indicated, as per gastroenterology MDT, and a high-grade lymphoma was felt to be the most likely diagnosis. There was a fear that the longer it took for a definitive lymphoma diagnosis to be reached, the less fit for therapy the patient would become, as his deterioration continued. Thus, following discussion between the treating consultant, the patient and his family, systemic treatment with cyclophosphamide, vincristine and prednisolone (CVP) was commenced. The patient also received weekly rituximab and a two-week course of ganciclovir to treat EBV and CMV reactivations, respectively.

Following two cycles of chemotherapy, the patient clinically improved with interval response demonstrated on CT-TAP; EBV and CMV viral loads decreased with treatment.

Biopsy of the mesenteric mass revealed a nodal PTCL of TFH origin (see below). Soon after his 3rd cycle of treatment, our patient was readmitted with pneumonia and progressive disease as seen on imaging. He continued to deteriorate despite treatment and following a discussion with the patient, supportive palliative care was pursued. He died shortly after, less than 6 months post initial presentation.

### Mesenteric Biopsy Results

Immunohistochemical (IHC) stains performed included CD3, CD20, CD5, CD10, BCL-2, CD30, ki67, CD68, CD2, CD7, CD56, CD10, CD79a, CD4, CD8, AE1/AE3, Cyclin D1 (Ventana), BCL-6, MUM1, HHV-8, PD-1, ICOS, CD21, CD23 (Cell Marque), TIA-1 (BioGenex) and in situ hybridisation (ISH) for EBER (Ventana), kappa and lambda (Roche).

**Figure 2:**
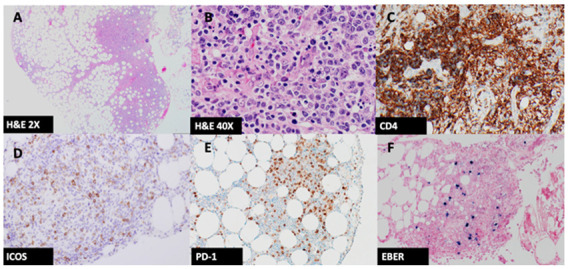
(**A**) High and (**B**) low power H&E images of the mesenteric biopsies showing an infiltrate of small- to medium-sized lymphoid cells with scattered blast cells, plasma cells and histiocytes (2× & 40× magnification, respectively); (**C**) The lymphoid cells are CD4 positive ((**C**), 20×) and express (**D**) ICOS and (**E**) PD-1 (**D**,**E**) 20×); (**F**) EBER ISH stains scattered lymphocytes ((**F**), 20×).

H&E sections of the mesenteric mass showed adipose tissue with a patchy nodular lymphocytic infiltrate. The lymphocytes were small–medium sized with mild nuclear contour irregularities, inconspicuous nucleoli, and abundant pale cytoplasm. Some scattered germinal centres were identified. There were background blast cells, plasma cells and histiocytes (Figure 2).

There were about equal CD20+ B cells and CD3+ T cells. T cells showed no significant loss of pan T cell antigen expression and were a mixture of CD4+ and CD8+ cells. CD21 and CD23 showed intact follicular dendritic cell meshworks. Some CD4+ T cells were cytologically atypical and stained positive for PD-1 and ICOS (Figure 2). These positive cells were present within the follicles and surrounding residual follicles, as one sees in AITL, Pattern 1. EBER ISH highlighted scattered lymphocytes (Figure 2). Remaining IHC was non-contributory.

**Figure 3:**
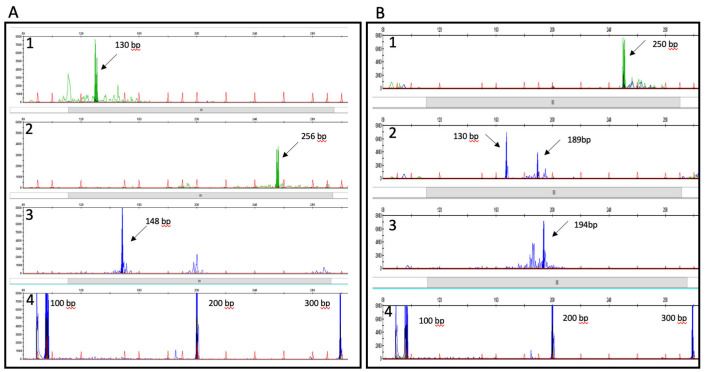
Immunoglobulin and T cell receptor gene rearrangement analysis. Genetic fragment analysis of immunoglobulin (Ig) (Panel (**A**)) and T cell receptor (TCR) gene rearrangements (Panel (**B**)) of tissue utilising Invivoscribe CE-IVD multiplex PCR Identiclone kits. Panel (**A**): 1. Ig Heavy chain (H) VFR3-JH; 2. IgH D1to6-JH; 3. Ig Kappa V-J; 4. DNA size quality. Panel (**B**): 1. TCR beta V-J1 + 2; 2. TCR beta D-J; 3. TCR gamma V-J; 4. DNA size quality. Arrows denote clonal rearrangements detected with these assays. Y axis = relative fluorescence units; X axis = DNA size in base pairs. Analysis software Genescan following capillary electrophoresis with Genescan 400HD ROX size standards on 3130XL analyser (Thermo Fisher, St. Louis, MO, USA).

Multiplex PCR showed clonal TCR beta and gamma gene rearrangements along with a clonal IgH gene rearrangement and a weak clonal IgK gene rearrangement (Supplementary) (Figure 3).

Next generation sequencing (NGS) (TruSight Oncology 500 Gene Panel) identified mutations in DNMT3A and TET2 consistent with a PTCL (Supplementary) [5]. A CARD11 mutation with a relatively low variant allele frequency (VAF) was also detected (Table 1).

Although the presentation of this case was highly atypical, examination of the morphology, the immunophenotype with an atypical CD4+ T cell infiltrate expressing two TFH markers (PD-1 and ICOS), as well as combining clonality and NGS results, a diagnosis of nodal PTCL of TFH origin was made.

## 3. Discussion

The 2016 revised WHO classification currently describes three lymphomas of TFH cell origin: AITL, FTCL and nodal PTCL with TFH phenotype. Despite morphological differences, immunophenotypic and molecular features overlap [6]. Our case meets the WHO criteria for diagnosis by atypical T cell infiltrate that is CD4+ and expressed two TFH markers (ICOS and PD-1), with the molecular studies being confirmatory (mutations in TET2 and DNMT3A). In the current WHO classification, this lymphoma would qualify as a nodal PTCL with TFH phenotype. These lymphomas, although not well defined, are nodal in origin and frequently show a diffuse infiltration pattern without morphological features seen in AITL, namely prominent polymorphic inflammatory background, vascular proliferation, and expansion of the FDC meshworks [7]. The absence of nodal involvement is highly unusual, as seen in our case. Additionally, our case differed with neoplastic cells representing a small proportion of the mass. In our case there was a large polymorphous inflammatory background including admixed B and T cells, plasma cells, blast cells, histiocytes as well as the presence of intact follicular dendritic meshworks.

Interestingly, some case reports have found transition from one TFH lymphoma to another in serial biopsies from the same patient suggesting they may represent different aspects of the same disease with morphological plasticity [8,9]. If TFH lymphomas represent the same common entity it is uncertain how our case fits into this spectrum.

Our case had clonal rearrangements of both TCR and immunoglobulin (IG) genes (Figure 3). Most AITLs have TCR gene rearrangements. Additionally, clonal IG gene rearrangements are detected in up to 50% which correlate, in part, with the expanded EBV+ B cells [10]. This may be a clue to diagnosis in these challenging cases, especially when morphology is suspicious but not diagnostic.

On a gene expression profiling level, lymphomas of TFH origin display distinctive gene signatures in both neoplastic and microenvironmental components which differ from PTCL-NOS. These tumours have been the object of multiple NGS studies which have revealed recurrent genetic mutations affecting TET2, DNMT3A, RHOA, and genes involved in TCR signalling pathways [11]. In TFH lymphomas, inactivating mutations in TET2 and DNMT3A, both epigenetic regulators, occur in both neoplastic T cells as well as admixed mature B cells, while RHOA and TCR signalling pathway gene mutations are restricted to the neoplastic T cells [12,13]. A multistep and multilineage tumorigenesis model has therefore been suggested with epigenetic dysregulation (loss-of-function mutations in DNMT3A and TET2) occurring as an early event in haematopoietic stem cells. This alone is not sufficient to drive lymphomagenesis and requires a second hit [14]. This second hit, usually at much lower allelic fractions due to its restriction to the neoplastic T cells, is usually in genes important for T cell function—either in RHOA^G17V^, seen in up to 70% of cases or in one of the genes involved in TCR signalling pathways (PLCG1, CD28, FYN) seen in 49% of cases [3,15]. Transcriptional profiling suggests gain-of-function mutations in these genes results in NF-kB activation [16].

Due to the high frequency of RHOA^G17V^ mutations in TFH lymphomas, it is a useful adjunct to support diagnosis. This mutation was not detected in our case. A subclonal CARD11^F902C^ mutation was identified (Table 1). CARD11 is a gene involved in TCR signalling. It encodes the caspase recruitment domain-containing protein required for CD28/TCR-induced NF-kB activation, and CARD11^F902C^ has been reported as a gain-of-function mutation in AITL [3]. In the absence of RHOA^G17V^ mutation, CARD11^F902C^ may constitute a ‘second hit’ that plays a role in this neoplasm. However, unequivocal determination of what cells this mutation is found in will require additional studies such as single-cell sequencing.

Clinically, what significance does this diagnosis have? Like PTCL-NOS, these patients typically have an aggressive clinical course, respond poorly to conventional treatments, and have a dismal prognosis, as seen in our case. However, accurately dividing lymphomas by their cell of origin will refine the molecular classification, improve prognostication, and help identify the most appropriate candidate therapeutic targets, as seen in many B cell lymphomas.

## 4. Conclusions

Here, we discuss an unusual case of a nodal PTCL with a TFH phenotype presenting as an isolated mesenteric mass with no nodal involvement which has never been described. This case highlights the diagnostic challenge posed by these rare lymphomas and how corresponding clinical findings, morphology, immunophenotypic findings, as well as molecular/genetic studies are all essential for reaching an accurate diagnosis.

## Figures and Tables

**Table 1:** Pathogenic mutations identified by NGS.

Small Nucleotide Variants			
**Gene**	**Transcript**	**Amino Acid Change**	VAF * (%)
**DNMT3A**	chr2:25457242	p.Arg882His	25%
**TET2**	chr4:106158236	p.Thr1047fs*9	21%
**TET2**	chr4:106193794	p.Leu1420fs*6	25%
**CARD11**	chr7:2955005	p.Phe902Cys	3.78%

* VAF: Variant Allele Frequency.

## Supplementary Materials

The following supporting information can be downloaded at https://www.mdpi.com/article/10.3390/hematolrep14040047/s1.
