# Supplementary material for: An Isolated Mesenteric Presentation of a Nodal Peripheral T Cell Lymphoma with T Follicular Helper Cell Phenotype
Source: Hematol Rep. 2022 Nov 15;14(4):335–41. doi: 10.3390/hematolrep14040047 (PMC9680281; doi:10.3390/hematolrep14040047)
Supplement: Supplementary file 1 [file hematolrep-14-00047-s001.zip › hematolrep-1872329-supplementary.pdf]

Supplementary Data

Materials and Methods: The mesenteric biopsy was fixed in 10% neutral-buffered formalin followed by parrafin embedding. Immunohistochemistry was performed routinely following standardised protocols. Immunohistochemical (IHC) stains performed included CD3, CD20, CD5, CD10, BCL-2, CD30, ki67, CD68, CD2, CD7, CD56, CD10, CD79a, CD4, CD8, AE1/AE3, Cyclin D1 (Ventana), BCL-6, MUM1, HHV-8, PD-1, ICOS, CD21, CD23 (Cell Marque), TIA-1 (BioGenex) and in situ hybridisation for EBER (Ventana), kappa and lambda (Roche). Multiplex polymerase chain reaction (PCR) analysis was performed using BioMED primers and probes with PCR products visualised using GeneScan as previously described [18]. For Next Generation Sequencing, nucleic acid was extracted from formalin-fixed paraffin-embedded tissue sections using the AllPrep DNA/RNA FFPE Kit. Libraries were prepared according to the instructions provided with the TruSight ®. Capture based next generation sequencing was performed using Oncology 500 (TSO500, Illumina) kit. Data Analysis Proprietary TruSight Oncology 500 v2.1 local applications (Illumina) were used for alignment, variant calling, ® gene fusion detection and for determination of TMB and MSI. All alignments are performed against Illumina designed browser extensible data (BED) files that are based on the human genome reference GRCh37/hg19 assembly. The resulting vcf file was uploaded to QIAGEN Clinical Insight (QCI) for variant filtering, variant annotation, variant classification, variant interpretation and reporting.

Reference

1. Van Dongen, J.J.; Langerak, A.W.; Brüggemann, M.; Evans, P.A.S.; Hummel, M.; Lavender, F.L.; Delabesse, E.; Davi, F.; Schuuring, E.; Garcia-Sanz, R.; et al. Design and standardization of PCR primers and protocols for detection of clonal immunoglobulin and T-cell receptor gene recombinations in suspect lymphoproliferations: Report of the BIOMED-2 Concerted Action BMH4-CT98-3936. *Leukemia* **2003**, *17*, 2257–2317. https://doi:10.1038/sj.leu.2403202.
